# Immunologic hypo- or non-responder in natural dengue virus infection

**DOI:** 10.1186/1423-0127-20-34

**Published:** 2013-05-31

**Authors:** Guey Chuen Perng, Kulkanya Chokephaibulkit

**Affiliations:** 1 Department of Microbiology and Immunology, Medical College, National Cheng Kung University, Tainan, 70101, Taiwan; 2 Center of Infectious Disease and Signaling Research, National Cheng Kung University, Tainan, 70101, Taiwan; 3 Department of Pathology and Laboratory Medicine, Emory Vaccine Center, Emory University School of Medicine, Atlanta, GA, 30322, USA; 4 Department of Pediatrics, Faculty of Medicine Siriraj Hospital, Mahidol University, Bangkok, 10700, Thailand

**Keywords:** Nonresponder, Naïve, Flavivirus, Dengue fever, DHF

## Abstract

Serologically defined primary dengue virus infection and/or subsequent homologous serotype infection is known to be associated with less severe disease as compared with secondary subsequent heterologous serotype infection. In geographical locales of high dengue endemicity, almost all individuals in the population are infected at some point in time and should therefore are at high risk of secondary infection. Interestingly, dengue viremia in healthy blood donors whose sera apparently lack detectable levels of specific antibody to dengue viral antigens has been reported. The incidence rate of potential immunologic hypo- or non-responders following natural primary dengue virus infection in dengue endemic regions, who do become immune responders only after repeated exposure, has not been described. These are the patients who may be diagnosed as primary infection in the subsequent infection, but actually are secondary infection. This concept has important implications with regards to the hypothesis of immunological enhancement of dengue pathogenesis, which has largely been advanced based on empirical observations and/or from in vitro experimental assays. The fact that dengue naïve travelers can suffer from severe dengue upon primary exposure while visiting dengue endemic countries underscores one of the major problems in explaining the role of immune enhancement in the pathogenesis of severe dengue virus infection. This evidence suggests that the mechanism(s) leading to severe dengue may not be associated with pre-existing enhancing antibody. Consequently, we propose a new paradigm for dengue virus infection classification. These include a) patients with naïve primary infection, b) those that are serologically defined primary in dengue endemic zones and c) those who are serologically defined secondary dengue virus infection. We submit that clarity with regards to such definitions may help facilitate the delineation of the potential mechanisms of severe dengue virus infection.

## Review

Dengue is one of the most important vector-borne human diseases globally as well as a major public health burden and threat. There are four distinct viral serotypes, each one of them is capable of causing a wide spectrum of dengue manifestations including plasma leakage and shock with multi-organ failure. The resurgence of the dengue endemnicity has resulted from numerous oscillating environmental, social and economical factors. Two-fifths of the world’s population is at risk of dengue virus infection, with approximately one-half million requiring hospitalization, with an estimated 25,000 deaths annually, according to the WHO. Currently, there are no effective antiviral modalities and/or preventive vaccines available to combat or control dengue virus infection. The precise mechanism by which only a small percentage of dengue virus infected individuals progessing to severe dengue disease remains poorly understood.

The pathophysiology of severe dengue virus infection is very complex and may involve multiple factors. One of the factors believed to play a role in the pathogenesis of severe dengue disease is the presence of pre-existing dengue reactive antibody as available data from dengue epidemic countries have indicated that severe disease more frequently occurs during subsequent viral infections with a different dengue serotype [1,2], as defined by the standard serological test. However, recent results obtained from non-dengue endemic regions [3] and from travelers suggest that the frequency of severe dengue diseases during primary infection in immune-naive individuals is similar to that of heterologous secondary infections in endemic areas [4]. The immune enhancement theory is further put to question by the study by Libraty et al [5] which included a cohort study that revealed the lack of an association between maternal antibodies and development of severe dengue in infected infants. Collectively, the evidence suggests that as yet undefined factor(s) play a critical role in the development of severe dengue in naïve primary infection. We submit that the cause of severe pathology in truly naïve individuals infected by dengue virus may be distinguishable from that of serologically defined primary infection in dengue endemic zones.

According to the WHO guidelines, it is required that paired specimens from individual patients be simultaneously processed to clearly define the infection as primary or secondary in dengue endemic regions. But, very often, paired-sample collection is impractical in routine clinical practice. This limitation has led to the definition of primary and secondary infection in dengue endemic zones by the analysis of the ratio of IgM/IgG on a single sample; if the value is >1.2, then it is a primary infection, but if the value is ≤1.2, it is noted as a secondary infection. Epidemiologically, serological surveillance studies have revealed that about 85 to 95% of school-aged children in endemic countries are positive for dengue IgG antibody [2,6,7]. Interestingly, a recent report [8] demonstrates that dengue viremia can exist in healthy blood donors whose sera apparently lack detectable levels of specific antibody to dengue virus (Table 1), and the incidence varies, ranging from 0.7/1000 to 4.5/1000, dependent upon season and year [9]. Thus, besides the use of the IgM/IgG ratio, it is difficult at best to distinguish between primary and secondary infection. It is further complicated by the incidence of non-classical serologic responses, in which the ratio value is often slightly below 1.2. Such cases are very often arbitrarily assigned as secondary infection, and thus the definition has been called into question [10]. The fact that there exist asymptomatic dengue viremia positive but antibody undetectable individuals in dengue endemic geographical locales, presents an important challenge to the blood supply of that region [11-13]. Dengue inapparent infection has been documented in literature since 1939, in which volunteers intravenously received serum taken from an acute dengue patient, but no clinical symptoms were observed, and yet serum taken from this subject was able to infect a new healthy volunteer. Hence the term dengue inapparent infection was instituted [14]. Interestingly, it has been proposed that asymptomatic dengue cases may account for the introduction and spread of dengue viruses in non-endemic regions [15]. Consequently, the cumulative evidence suggests that asymptomatic viremia may have an important role in dengue transmission and warrant a more in depth investigation.

**Table 1:** DENV detection in blood donations collected in Puerto Rico in 2007

	S/CO by TMA*		**S/CO by eTMA**		**CDC Testing**			
**Unit**	**Initial**	**Retest**	**Initial**	**Retest**	**Serotype**^**$**^	**Viral load (Copies/ml)**	**C6/36**^**#**^	**Anti-DENV IgM**
1	**27.75**	**38.99**	**87.16**	**88.52**	DENV-2	1.12 × 109	**Pos**	Neg
2	**32.34**	**33.30**			DENV-2	5.08 × 108	**Pos**	**Pos**
3^&^	**33.30**	**37.38**	**91.10**	**83.09**	DENV-2	1.35 × 108	**Pos**	Neg
4	**37.66**	**39.16**	**87.13**	**89.32**	DENV-3	7.25 × 107	**Pos**	Neg
5	**40.29**	**27.03**	**82.29**	**92.04**	DENV-3	1.37 × 107	**Pos**	Neg
6	**32.73**	**35.03**			DENV-3	1.18 × 107	**Pos**	Neg
7	**33.91**	**32.87**			DENV-3	7.67 × 106	**Pos**	Neg
8	**31.97**	**30.59**			DENV-1	4.49 × 106	**Pos**	Neg
9	**19.14**	**13.94**			DENV-2	2.82 × 106	**Pos**	**Pos**
10	**33.10**	**38.68**	**87.86**	**89.91**	DENV-3	6.39 × 105	**Pos**	Neg
11	**31.25**	**33.56**			DENV-3	3.50 × 105	**Pos**	Neg
12	**5.68**	**20.55**	**29.48**	**21.59**	DENV-3	1.00 × 105	**Pos**	Neg
13	**34.81**	**37.21**	**76.16**	**32.72**		<103	Neg	Neg
14	**23.38**	**31.07**	**31.25**	**31.18**		<103	Neg	Neg
15	**14.23**	**23.26**	**28.59**	**3.28**		<103	Neg	**Pos**
16	**13.14**	**25.77**	**29.26**	**12.51**		<103	Neg	Neg
17	**11.51**	**5.63**				<103	Neg	Neg
18	**8.17**	**16.58**				<103	Neg	Neg
19	**6.64**	**8.91**				<103	Neg	**Pos**
20	**5.06**	**4.12**	**29.96**	**8.61**		<103	Neg	Neg
21	**3.37**	**4.95**				<103	Neg	**Pos**
22	**2.95**	**25.28**				<103	Neg	**Pos**
23	**8.20**	**1.40**				<103	Neg	Neg
24	**4.46**	0.01	**24.80**	0.06		<103	Neg	Neg
25	**1.02**	**2.29**	**28.01**	0.01		<103	Neg	Neg
26^%^	0.45		**26.38**	**27.55**		<103	Neg	Neg
27^%^	0.17		**26.18**	**30.99**		<103	Neg	Neg
28^%^	0.30		**25.31**	**29.11**		<103	Neg	Neg
29^%^	0.50		**24.34**	**17.85**		<103	Neg	Neg

*TMA reactive when the S/CO ratio is 1.00 or greater. ^$^Serotype-specific, real-time RT-PCR. ^#^C6/36=the mosquito cell line used for infectivity studies. ^&^Unit3 was involved in a transfusion transmission. ^%^Four TMA nonreactive samples were eTMA reactive. Bold text indicates positive values. With permission from originally published in Transfusion 2012 Aug; 52(8):1657-66.

**Figure 1:**
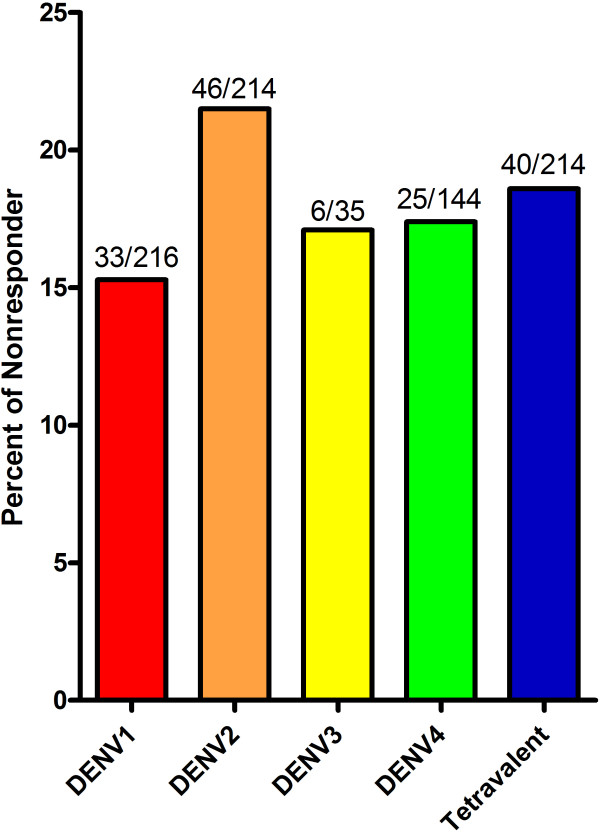
**Frequency of immunologic non-responders derived from vaccinees received one dose of monovalent or tetravalent dengue vaccines.** Cumulative data were gathered from numerous resources with complete information given in dengue vaccine clinical trials [35-52]. The frequency of immunologic non-responders is inferred from the rate of seroconversion after one dose in these vaccinees.

Within the context of diagnoses that are based on serological tests, it is important to note the results that have been recorded following routine vaccinations. The estimated frequency of immunologic non-responders among Hepatitis B vaccine and Venezuelan equine encephalitis (VEE) vaccine recipients is around 1-10% [16-18] and about 18-26% [19], respectively. However, the occurrence of such non-responders following natural Hepatitis B or VEE infection has not so far been investigated or documented. Furthermore, although the frequency of hypo- or non-responders in natural dengue virus infection has never been explored, recent data accumulated from screening of healthy blood donors living in a dengue endemic zone suggest that the incidence ranges from 4.5 to 0.7 per 1000 donors [9]. In addition, extrapolation of cumulative data of seroconversion following monovalent or tetravalent dengue vaccine trials suggested that hypo- or non-responders after a single dose of vaccine was found to be between 15 to 22% (Figure 1), regardless where the trials are performed. Epidemiologically, the prevalence of IgM and/or IgG negative individuals reported in serological studies conducted in dengue endemic regions indicates that the range is 1 to 10% among populations in various geographical areas [6,7,20-26]. These data suggest that subjects that are initially immune non-responders may be re-challenged repeatedly with the incoming virus throughout their lives in endemic countries. Interestingly, it has been documented that the frequency of reattack rate in dengue endemic regions or during an outbreak is about 1:20, or 5-8% [27-32]. Coincidentally, the seroconversion rate in dengue vaccine trials can be up to 90 to 99% after the 2^nd^ and 3^rd^ dose [33,34]. Consequently, several pragmatic questions can be asked; should immunologic non-responders after being re-challenged be defined as primary or secondary infection in dengue endemic regions? Will these non-responders develop severe dengue after repeated exposures with the virus in the natural setting? These questions should be thoughtfully considered and investigated.

One of avenues that can be linked to the immune non-responder is genetic polymorphism in ethnic origin of populations and perhaps in a specific individual. In a general term, immune related genetic polymorphisms are much more likely contributing to the phenomena. These include genes for human leukocyte antigens (HLA) or major histocompatibility class, for antibody genes, for cytokines, for Toll-like receptors or receptors in pathogen pattern recognition and others [53,54]. Some of these genetic polymorphisms have been associated with quality of immune response and viral disease development, as well as that found in individuals who are hypo- or non-responders to hepatitis B vaccination [55-62]. Recent evidence suggests that defective in antigen intake by the antigen-presenting cells and/or unable to present antigen properly and adequately is not a cause of non-responsiveness to hepatitis B surface antigen from HLA polymorphism [63]. Scientifically, the cascade and complexity of immune responses in pathogen- or vaccine-induced can be highly variable among individuals. Consequently, any genes within the immune response network would potentially contribute to the pleiotropic variation seen in an infected population or vaccinees.

As for how to differentiate the naïve primary infection from hypo/non-responder when no host gene biomarkers are currently available, a number of methods have been proposed. These include a) analysis of an individual’s genetic background and HLA typing, b) performing a much more laborious laboratory test such as the identification of a molecular signature using whole genome transcriptional analysis in PBMC [64] or c) the assessment of CD4^+^ T cell subsets, CD31^+thymic^ naïve CD4+ T cells, a prognostic marker for immune competence [65]. Thus, in order to further advance the understanding of the pathogenesis of severe dengue, three major categories, naïve primary infection, serologically defined primary infection in endemic zones, and secondary infection, are suggested and should be implemented where it is applicable in the interpretation of results.

The presence of different dengue serotypes and a sub-genotype group within a serotype are the well-established complexity in dengue. Each serotype or sub-genotype is capable of inducing typical dengue diseases. Although some sub-genotypes may induce severe dengue more often than others [66,67], dependent upon geographical zones, the concept on the immune nonresponder in dengue endemic regions has not been established in spite of epidemiologically results indicate that repeating exposure with alternate viral serotype correlates with dengue severity [68]. Consequently, in dengue endemic zones, is severe dengue a result of alternate heterologous infection in the prior exposure individual or a non-responder constantly re-challenged by circulating virus remains to be investigated. Currently, there is no assay that can differentiate the sequence of serotype infections in an individual [69]. Therefore, diagnostic tool that can efficiently differentiate the previous infected serotype prior to current other serotype infection is urgently needed.

## Conclusion

The ability to identify and distinguish these 3 categories will shed new light on the development of better diagnostic tools, mitigation of the threat to the blood supply in dengue-endemic countries, and pave a new avenue for molecular processes of immune development in the design and generation of modern vaccines. Furthermore, with a clearer definition of the virus pre-exposure, the search for better diagnostic marker and the identity of the pathogenic cause for severe dengue may be much simpler and faster to reach a consensus which would greatly facilitate the institution of effective and appropriate preventive medicine strategy.

## Competing interests

The authors declare that they have no competing interests.

## Authors’ contributions

GCP collected information, designed and organized the structure of the contents and wrote the manuscript. KC reviewed literature, discussed and suggested the contents as well as edited the manuscript. Both authors read and approved the final manuscript.
